# Supplementary material for: Illegitimate Recombination between Duplicated Genes Generated from Recursive Polyploidizations Accelerated the Divergence of the Genus Arachis
Source: Genes (Basel). 2021 Dec 1;12(12):1944. doi: 10.3390/genes12121944 (PMC8701993; doi:10.3390/genes12121944)
Supplement: Supplementary file 1 [file genes-12-01944-s001.zip › Supplementary Files/Supplemental figures S1-S12 and tables S1-S16.pdf]

genes

Supplemental information:

Illegitimate recombination between duplicated genes generated from recursive polyploidizations accelerated the divergence of genus *Arachis*

Shaoqi Shen[1, †], Yuxian Li[1, †], Jianyu Wang[1], Chendan Wei[1], Zhenyi Wang[1], Weina Ge[1], Min Yuan[1], Lan Zhang[1], Li Wang[1], Sangrong Sun[1], Jia Teng[1], Qimeng Xiao[1], Shoutong Bao[1], Yishan Feng[1], Yan Zhang[1], Jiaqi Wang[1], Yanan Hao[1], Tianyu Lei[1 *], Jinpeng Wang[1,2,3, *]

[1]School of Life Sciences, and Center for Genomics and Computational Biology, North China University of Science and Technology, Tangshan, Hebei 063000, China

[2]University of Chinese Academy of Sciences, Beijing 100049, China

[3]State Key Laboratory of Systematic and Evolutionary Botany, Institute of Botany, Chinese Academy of Science, Beijing 100093, China

[†]These authors contributed equally to this work.

[*]Address correspondence to Jinpeng Wang (Email: wangjinpeng@ibcas.ac.cn) and Tianyu Lei (Email: leitianyu@ncst.edu.cn).

Supplemental figures:

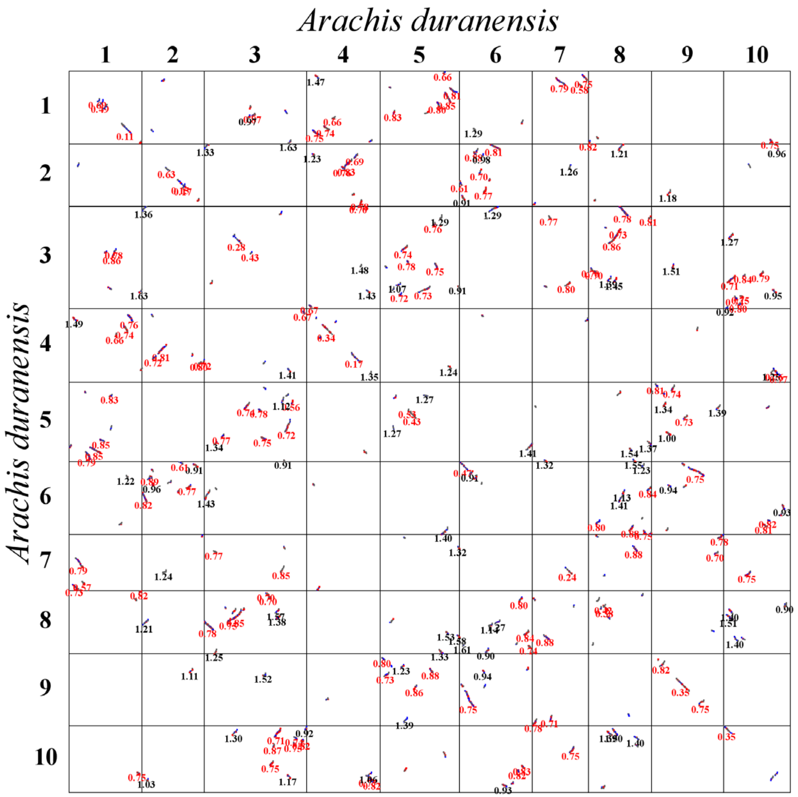

Supplemental Figure S1. The homologous colinearity *Ks* dotplot within *A. duranensis* genomes. The best, secondary, and other matched homologous gene pairs were shown by red, blue and gray dots, respectively. Mean *Ks* of each inferred colinear blocks are shown besides. The *Ks* value <=0.90 are in red, and others in black, often showing paralogous blocks in duplication events.

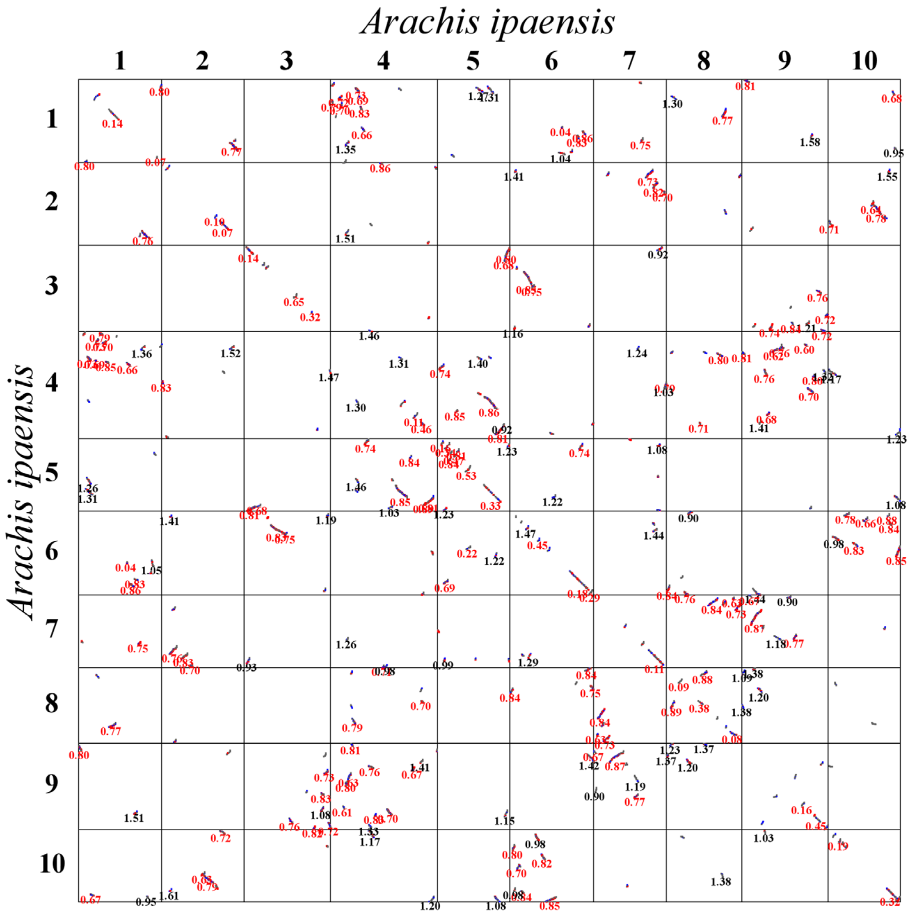

Supplemental Figure S2. The homologous colinearity *Ks* dotplot within *A. ipaensis* genomes. The best, secondarily, and other matched homologous gene pairs were shown by red, blue and gray dots, respectively. Mean *Ks* of each inferred colinear blocks are shown besides. The *Ks* value <=0.90 are in red, and others in black, often showing paralogous blocks in duplication events.

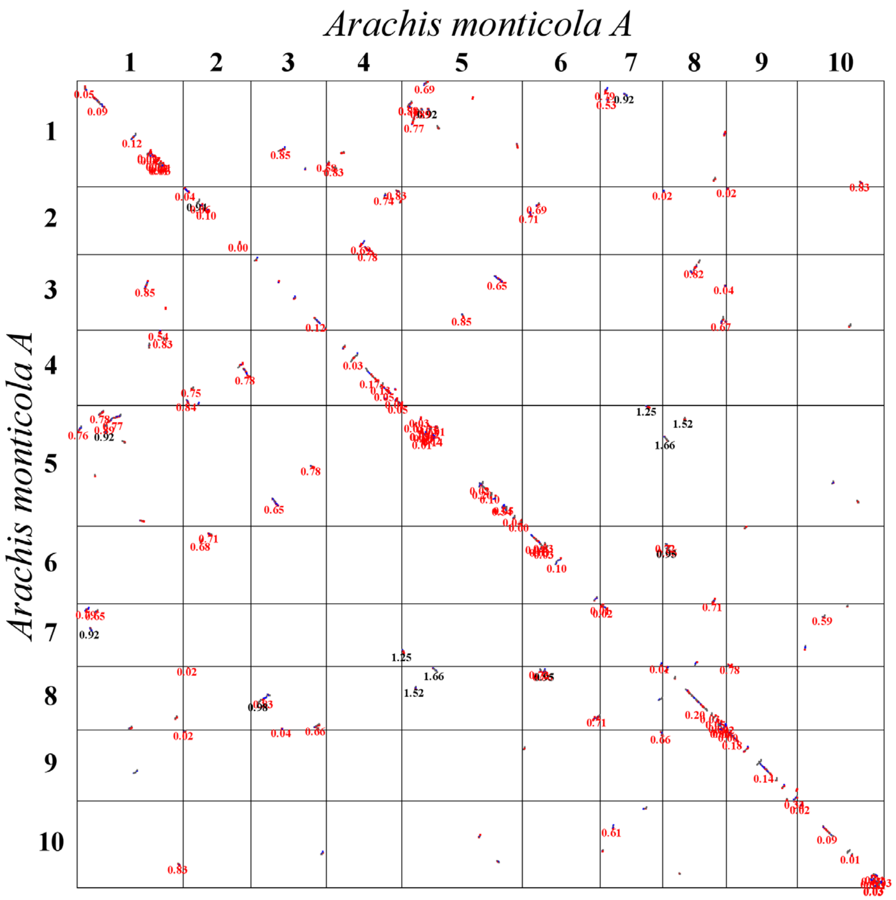

Supplemental Figure S3. The homologous colinearity *Ks* dotplot within *A monticola* A genomes. The best, secondarily, and other matched homologous gene pairs were shown by red, blue and gray dots, respectively. Mean *Ks* of each inferred colinear blocks are shown besides. The *Ks* value <=0.90 are in red, and others in black, often showing paralogous blocks in duplication events.

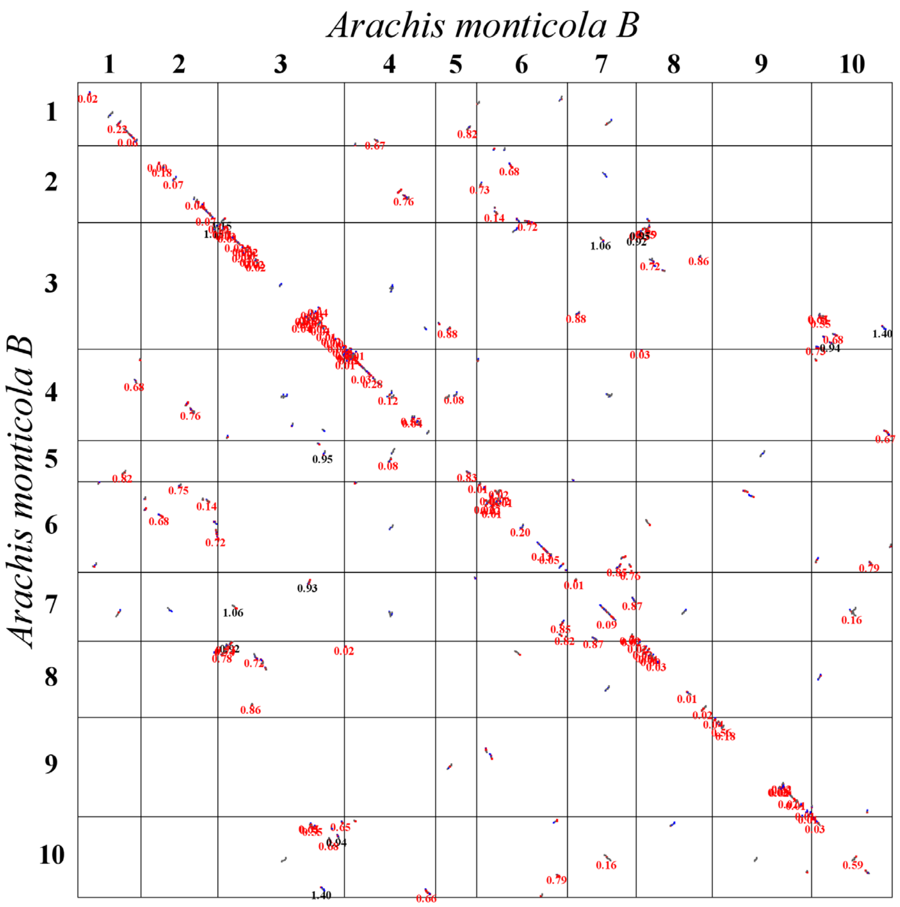

Supplemental Figure S4. The homologous colinearity *Ks* dotplot within *A monticola* B genomes. The best, secondarily, and other matched homologous gene pairs were shown by red, blue and gray dots, respectively. Mean *Ks* of each inferred colinear blocks are shown besides. The *Ks* value <=0.90 are in red, and others in black, often showing paralogous blocks in duplication events.

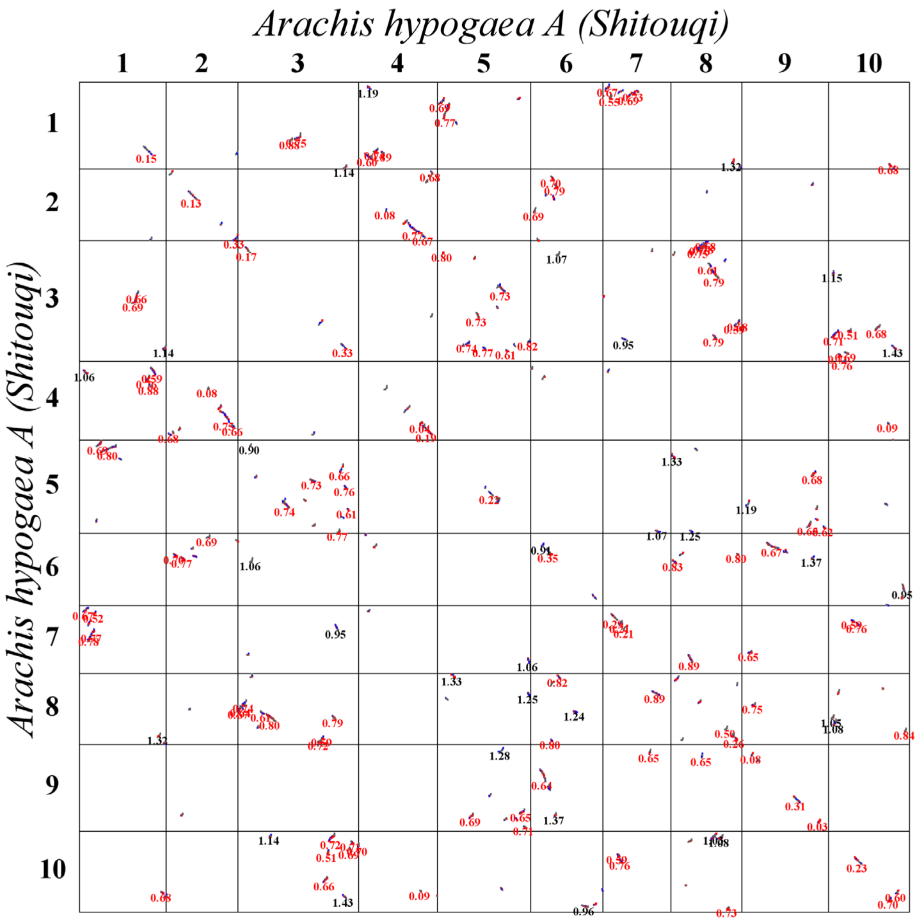

Supplemental Figure S5. The homologous colinearity *Ks* dotplot within *A. hypogaea* A (Shitouqi) genomes. The best, secondarily, and other matched homologous gene pairs were shown by red, blue and gray dots, respectively. Mean *Ks* of each inferred colinear blocks are shown besides. The *Ks* value <=0.90 are in red, and others in black, often showing paralogous blocks in duplication events.

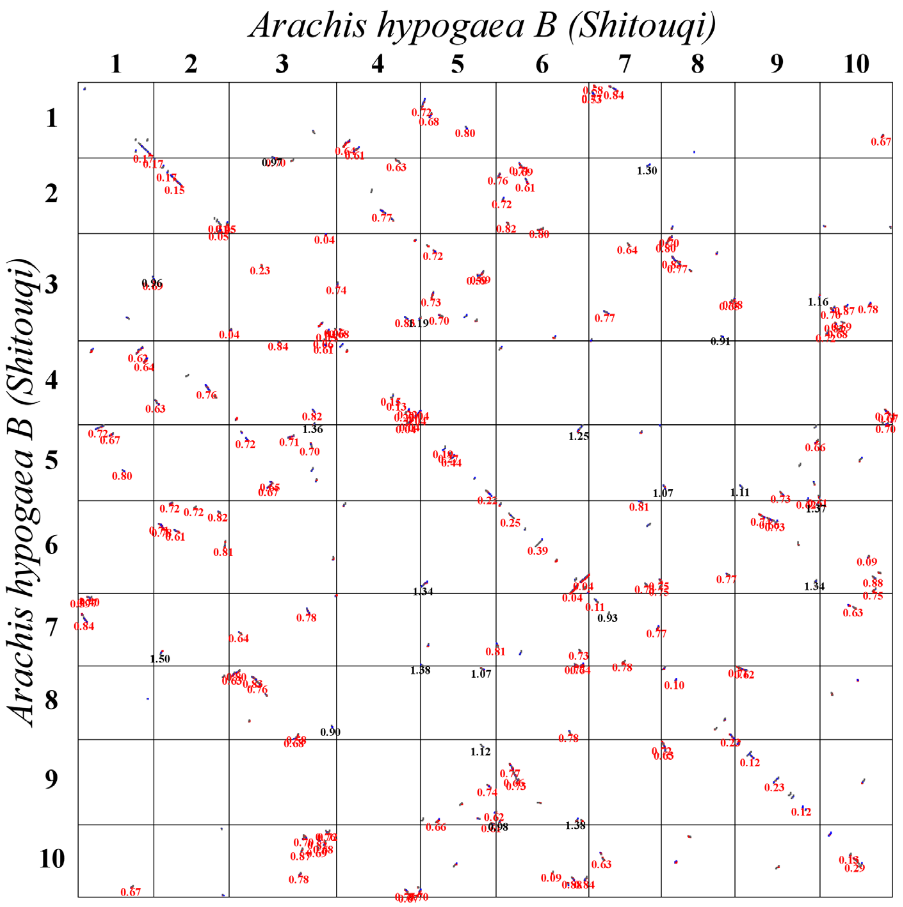

Supplemental Figure S6. The homologous colinearity *Ks* dotplot within *A. hypogaea* B (Shitouqi) genomes. The best, secondarily, and other matched homologous gene pairs were shown by red, blue and gray dots, respectively. Mean *Ks* of each inferred colinear blocks are shown besides. The *Ks* value <=0.90 are in red, and others in black, often showing paralogous blocks in duplication events.

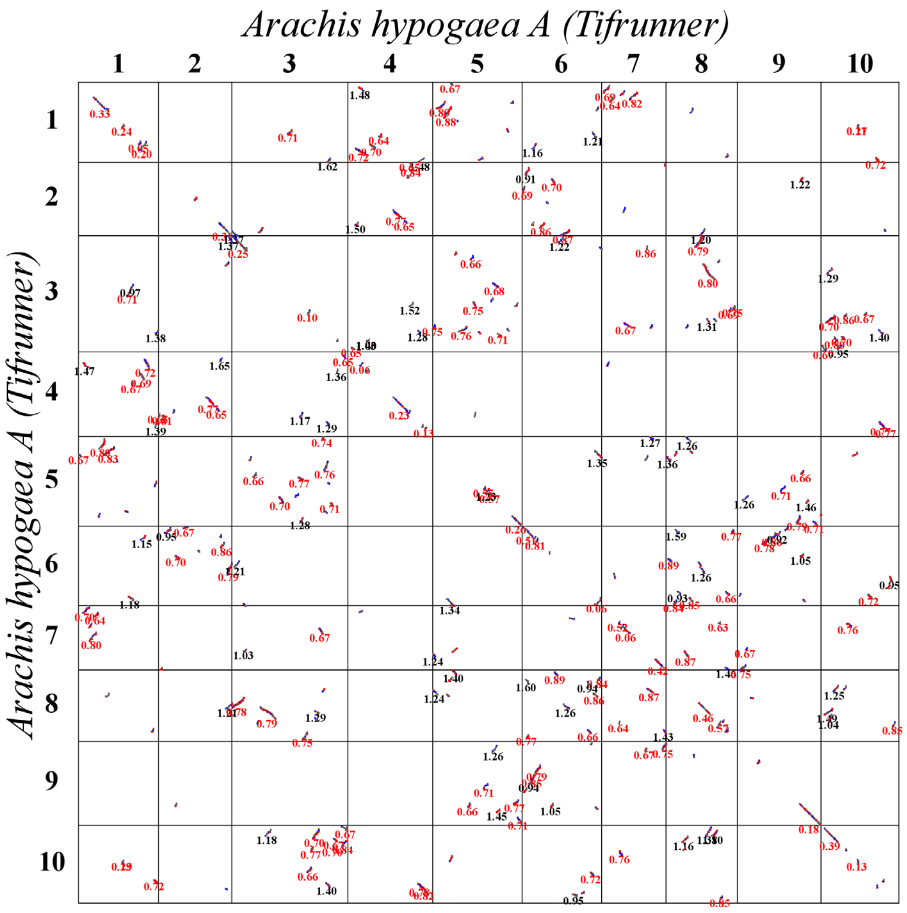

Supplemental Figure S7. The homologous colinearity *Ks* dotplot within *A. hypogaea* A (Tifrunner) genomes. The best, secondarily, and other matched homologous gene pairs were shown by red, blue and gray dots, respectively. Mean *Ks* of each inferred colinear blocks are shown besides. The *Ks* value <=0.90 are in red, and others in black, often showing paralogous blocks in duplication events.

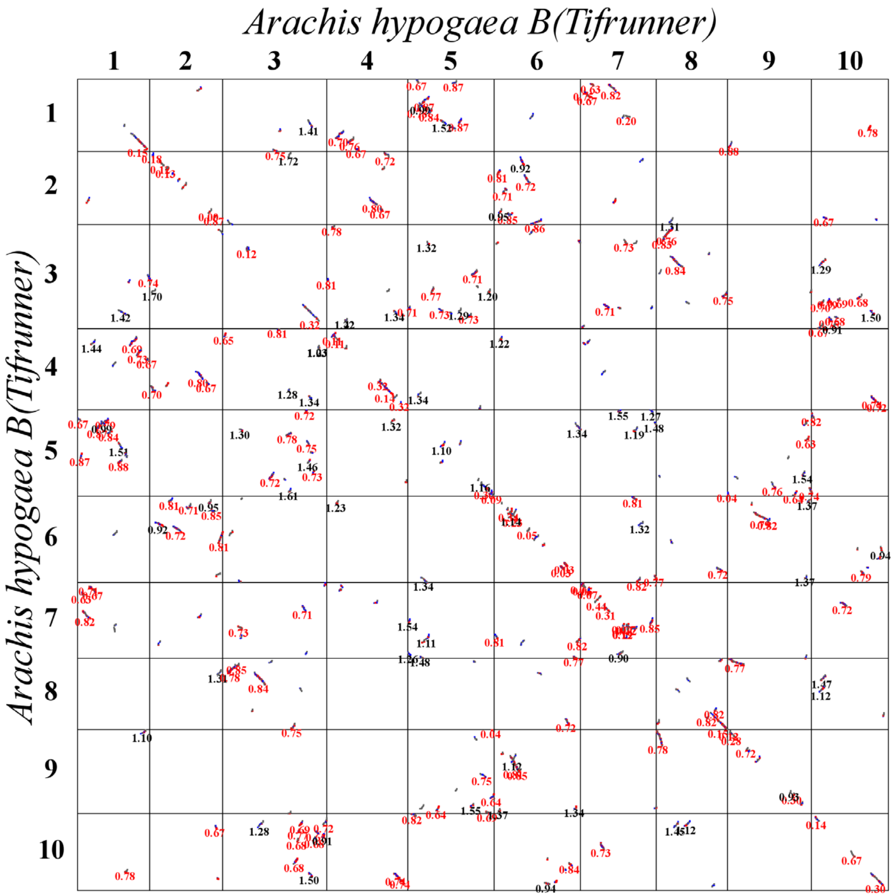

Supplemental Figure S8. The homologous colinearity *Ks* dotplot within *A. hypogaea* B (Tifrunner) genomes. The best, secondarily, and other matched homologous gene pairs were shown by red, blue and gray dots, respectively. Mean *Ks* of each inferred colinear blocks are shown besides. The *Ks* value <=0.90 are in red, and others in black, often showing paralogous blocks in duplication events.

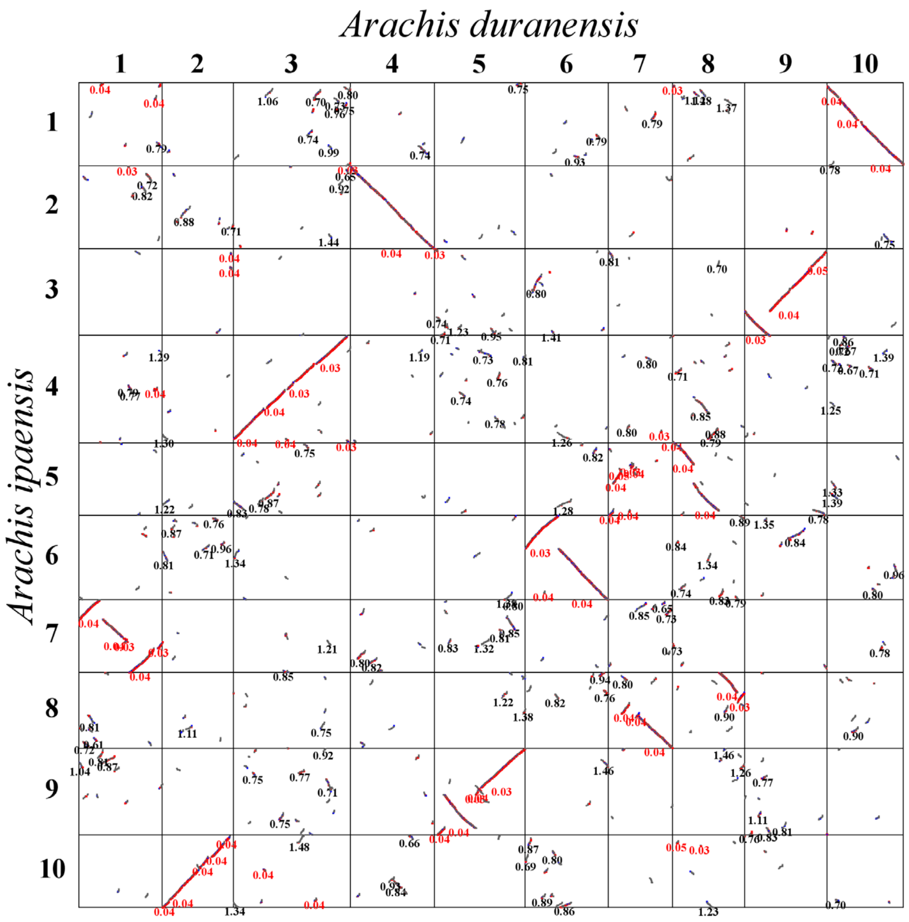

Supplemental Figure S9. The homologous colinearity *Ks* dotplot between *A. ipaensis* and *A. duranensis* genomes. The best, secondarily, and other matched homologous gene pairs were shown by red, blue and gray dots, respectively. Mean *Ks* of each inferred colinear blocks are shown besides. The *Ks* value <=0.10 are in red, and others in black, often showing paralogous blocks in duplication events.

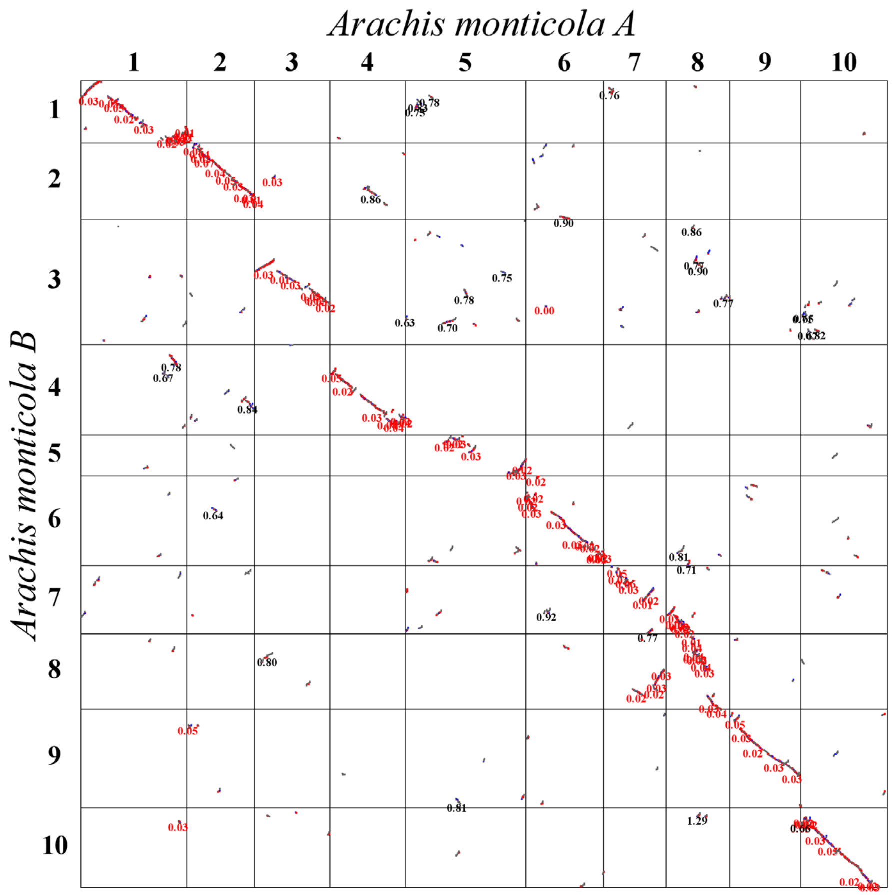

Supplemental Figure S10. The homologous colinearity *Ks* dotplot between *A monticola* A and *A monticola* B genomes. The best, secondarily, and other matched homologous gene pairs were shown by red, blue and gray dots, respectively. Mean *Ks* of each inferred colinear blocks are shown besides. The *Ks* value <=0.10 are in red, and others in black, often showing paralogous blocks in duplication events.

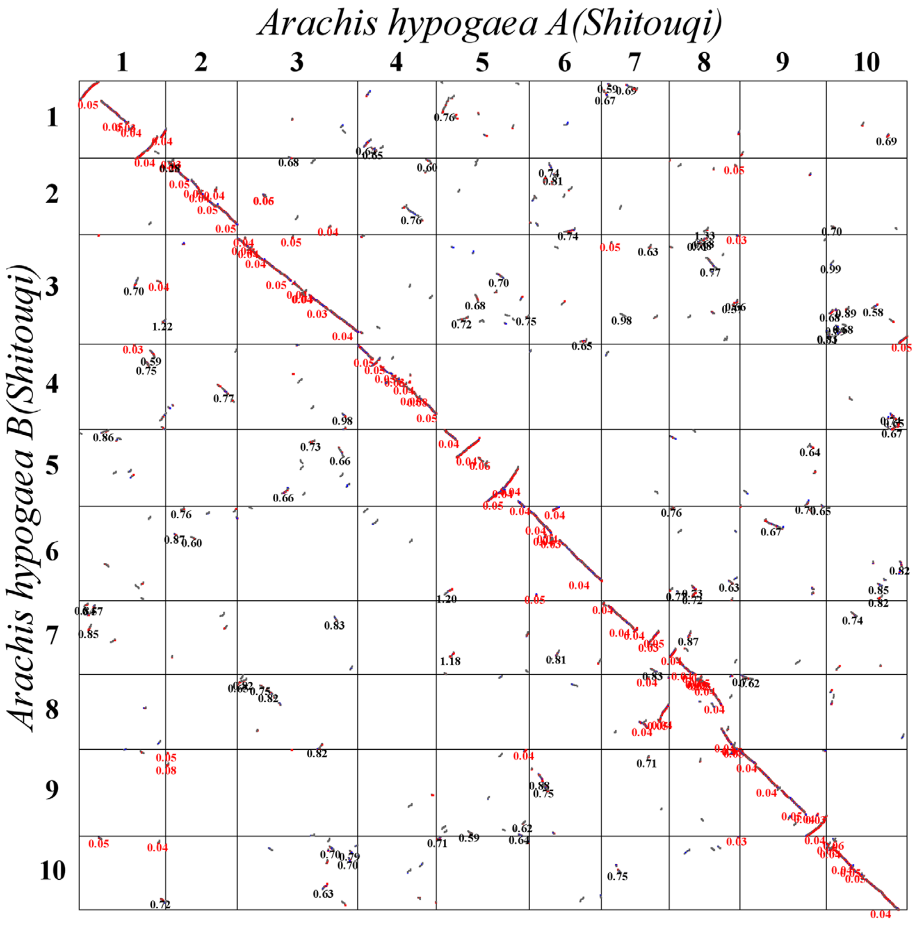

Supplemental Figure S11. The homologous colinearity *Ks* dotplot between *A. hypogaea* A (Shitouqi) and *A. hypogaea* B (Shitouqi) genomes. The best, secondarily, and other matched homologous gene pairs were shown by red, blue and gray dots, respectively. Mean *Ks* of each inferred colinear blocks are shown besides. The *Ks* value <=0.10 are in red, and others in black, often showing paralogous blocks in duplication events.

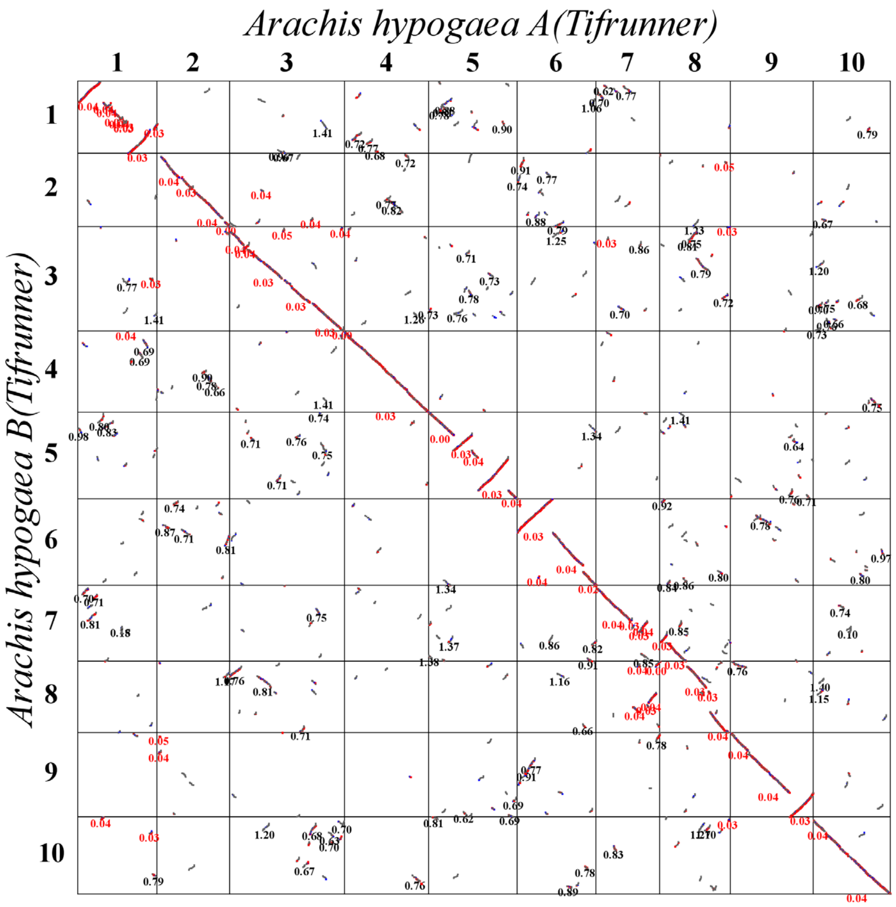

Supplemental Figure S12. The homologous colinearity *Ks* dotplot between *A. hypogaea* A (Tifrunner) and *A. hypogaea* B (Tifrunner) genomes. The best, secondarily, and other matched homologous gene pairs were shown by red, blue and gray dots, respectively. Mean *Ks* of each inferred colinear blocks are shown besides. The *Ks* value <=0.10 are in red, and others in black, often showing paralogous blocks in duplication events.

Supplemental tables:

Supplemental Table S1. Number of homologous blocks and gene pairs within a genome or between genomes.

Homologous Blocks within and among genome	BL[a] > 4	BL >10	BL >20	BL >50	ACGP[b]	LDB[c]	LDB on chromosomes
A. duranensis	5,016/599	2,355/106	1,517/40	554/8	8.37, 22.22, 37.92, 69.25	110	Ad03-Ad08
A. ipaensis	5,218/635	2,380/106	1,531/40	499/7	8.22, 22.45, 38.27, 71.29	85	Ai04-Ai05
A. monticola A	2,785/431	759/45	351/12	-/-	6.46, 16.87, 29.25, -	42	Ama01-Ama05
A. monticola B	3,561/570	742/51	187/7	-/-	6.25, 14.55, 26.71, -	41	Amb03-Amb08
A. hypogaea A (Shitouqi)	3,914/548	1,332/70	697/21	169/3	7.17, 19.03, 33.19, 56.33	61	Aha03-Aha10
A. hypogaea B (Shitouqi)	4,664/556	2,150/89	1,414/33	585/5	8.39, 24.16, 42.85, 117.00	195	Ahb06-Ahb06
A. hypogaea A (Tifrunner)	4,617/580	1,981/95	1,157/31	362/5	7.96, 20.85, 37.32, 72.40	95	Aha03-Aha08
A. hypogaea B (Tifrunner)	5,045/665	2,031/97	1,238/36	363/5	7.59, 20.94, 34.39, 72.60	86	Ahb03-Ahb08
A. duranensis vs A. ipaensis	37,293/2,802	24,457/337	22,074/151	19,458/65	13.31, 72.57, 146.19, 299.35	1,663	Ai02-Ad04
A. monticola A *vs A. monticola* B	15,894/2,170	5,828/214	4,065/81	2,565/29	7.32, 27.23, 50.19, 88.45	211	Amb10-Ama10
A. hypogaea A *vs A. hypogaea* B (Shitouqi)	31,851/2,991	18,070/310	15,884/143	13,392/61	10.65, 58.29, 111.08, 219.54	693	Ahb03-Aha03
A. hypogaea A *vs A. hypogaea* B (Tifrunner)	32,815/2,406	21,805/316	19,580/142	16,821/52	13.64, 69.00, 137.89, 323.48	1,613	Ahb04-Aha04

Note: [a]BL: block_length; [b]ACGP: average colinear gene pairs respectively per block; [c]LDB: number of colinear gene pairs reside in longest duplicated block.

Supplemental Table S2. Number of paralogous and orthologous gene pairs within genome or between studied genomes.

**Species**	***A. duranensis***	***A. ipaensis***	***A. monticola A***	***A. monticola* B**	***A. hypogaea* A (Shitouqi)**	***A. hypogaea* B (Shitouqi)**	***A. hypogaea* A (Tifrunner)**	***A. hypogaea* B (Tifrunner)**
A. duranensis	2,460	1,8264	10,292	9,730	16,784	14,424	17,981	15,987
A. ipaensis	18,264	2,203	9,144	11,489	14,091	15,897	15,678	20,245
A. monticola A	10,292	9,144	877	5,297	10,170	7,922	10,785	8,737
A. monticola B	9,730	11,489	5,297	972	8,504	11,932	9,166	12,089
A. hypogaea A (Shitouqi)	16,784	14,091	10,170	8,504	1,837	14,546	17,182	13,566
A. hypogaea B (Shitouqi)	14,424	15,897	7,922	11,932	14,546	1,970	13,886	19,264
A. hypogaea A (Tifrunner)	17,981	15,678	10,785	9,166	17,182	13,886	2,460	16,639
A. hypogaea B (Tifrunner)	15,987	20,245	8,737	12,089	13,566	19,264	16,639	2,203

Note: The diagonal lines represent the number of paralogous genes within species, while the others represent orthologous genes between species.

Supplemental Table S3. Gene conversion between PRT-related duplicated genes in *A. monticola* and *A. hypogaea* genomes.

Species	Quartets patterns	Homologous gene in Quartets	WCV-I[a]	WCV-II[b]	PCV[c]	Total	Conversion rate (%)
Arachis monticola	Ad-Ama-Ai-Amb	2,625	263	354	5	433	16.5%
Arachis hypogaea (Shitouqi)	Ad-Aha-Ai-Ahb	9,972	607	1,006	9	1,122	11.3%
Arachis hypogaea (Tifrunner)	Ad-Aha-Ai-Ahb	12,657	1,155	1,495	29	1,706	13.5%

Note: [a]WCV-I: The similarity of homologous gene pairs measured by *Ks*. [b] WCV-II: The ratios of amino acid locus identity of sequences in each quartet to measure the similarity, and examination of the topological tree changes. [c] PCV: A dynamic programming algorithm combined with phylogenetic analysis.

Supplemental Table S4. Nucleotide substitution rates of duplicate genes from quartets in studied peanut genomes.

Paralogues		Converted genes	Nonconverted genes	P-value (T- *test*)
A. duranensis	*Pn*	0.181	0.199	6.32×10^{-3}
	Ps	0.534	0.559	6.13×10^{-4}
	Pn/*Ps*	0.339	0.356	2.21×10^{-1}
A.ipaensis	*Pn*	0.184	0.202	4.38×10^{-3}
	Ps	0.542	0.560	1.27×10^{-2}
	Pn/*Ps*	0.339	0.361	1.20×10^{-2}
A.monticola A	*Pn*	0.200	0.210	7.69×10^{-1}
	Ps	0.557	0.555	9.35×10^{-1}
	Pn/*Ps*	0.359	0.378	7.81×10^{-1}
A.monticola B	*Pn*	0.234	0.204	3.51×10^{-1}
	Ps	0.566	0.532	9.30×10^{-1}
	Pn/*Ps*	0.413	0.383	3.75×10^{-1}

A.hypogaea A (Shitouqi)	*Pn*	0.182	0.271	1.12×10^{-8}
	Ps	0.528	0.586	1.97×10^{-7}
	Pn/*Ps*	0.345	0.462	$1.06\ 10^{-6}$
A.hypogaea B (Shitouqi)	*Pn*	0.183	0.285	5.45×10^{-10}
	Ps	0.528	0.594	1.92×10^{-8}
	Pn/*Ps*	0.347	0.480	5.45×10-8
A.hypogaea A (Tifrunner)	*Pn*	0.176	0.241	7.97×10^{-7}
	Ps	0.526	0.574	3.71×10^{-6}
	Pn/*Ps*	0.335	0.420	2.29×10^{-4}
A.hypogaea B (Tifrunner)	*Pn*	0.201	0.250	6.10×10^{-5}
	Ps	0.547	0.580	6.54×10^{-4}
	Pn/*Ps*	0.367	0.431	4.69×10^{-4}

Supplemental Table S5. Nucleotide substitution rates of PRT-related duplicated genes from quartets between peanut genomes.

Homologous genes		Converted genes	Nonconverted genes	P-value (T- *test*)
A. monticola	*Pn*	0.0233	0.0453	3.71×10^{-8}
	Ps	0.0552	0.100	3.40×10^{-8}
	Pn/*Ps*	0.422	0.453	2.57×10^{-7}
A. hypogaea (Shitouqi)	*Pn*	0.0197	0.0385	3.14×10^{-17}
	Ps	0.0528	0.0788	1.05×10^{-12}
	Pn/*Ps*	0.373	0.489	1.85×10-3
A. hypogaea (Tifrunner)	*Pn*	0.0145	0.0256	1.62×10^{-14}
	Ps	0.0371	0.0627	1.33×10^{-21}
	Pn/*Ps*	0.391	0.408	4.71×10^{-6}
A. duranensi-A.	*Pn*	0.0601	0.0250	5.29×10^{-23}

monticola A	*Ps*	0.126	0.0449	1.88×10^{-32}
	Pn/*Ps*	0.477	0.558	2.02×10^{-8}
A. ipaensis-*A.* *monticola* B	*Pn*	0.0495	0.0243	8.08×10^{-13}
	Ps	0.0962	0.0402	2.25×10^{-18}
	Pn/*Ps*	0.515	0.604	1.33×10^{-9}
A. duranensi-*A.* *hypogaea* A (Shitouqi)	*Pn*	0.0844	0.0207	3.13×10^{-262}
	Ps	0.146	0.0343	4.35×10^{-272}
	Pn/*Ps*	0.578	0.603	9.83×10^{-9}
A. ipaensis-*A.* *hypogaea* B (Shitouqi)	*Pn*	0.0855	0.0273	4.07×10^{-125}
	Ps	0.145	0.0424	1.23×10^{-129}
	Pn/*Ps*	0.590	0.644	6.82×10^{-23}
A. duranensi-*A.* *hypogaea* A (Tifrunner)	*Pn*	0.0590	0.0155	7.04×10^{-217}
	Ps	0.105	0.0280	9.40×10^{-218}
	Pn/*Ps*	0.562	0.554	8.70×10^{-10}
A. ipaensis-*A.* *hypogaea* B (Tifrunner)	*Pn*	0.0618	0.0187	5.05×10^{-148}
	Ps	0.110	0.0318	1.29×10^{-133}
	Pn/*Ps*	0.562	0.588	3.86×10^{-3}

Supplemental Table S6. The conversion rate of LCT-related duplicated genes and physical location of genes on chromosomes.

Species		Chr1	Chr2	Chr3	Chr4	Chr5	Chr6	Chr7	Chr8	Chr9	Ch10	All	P-value
Arachis duranensis	All converted	60 (14.1%)	26 (9.7%)	67 (10.4%)	37 (12.5%)	48 (10.3%)	50 (11.8%)	31 (13.8%)	48 (11.7%)	35 (13.2%)	38 (12.2%)	440 (11.8%)	0.998
	Paralogues	427	269	646	296	468	424	225	410	265	312	3,742	
Arachis ipaensi	All converted	59 (17.4%)	37 (12.1%)	29 (10.9%)	72 (11.2%)	46 (14.0%)	57 (13.4%)	57 (14.5%)	40 (13.5%)	45 (9.6%)	42 (15.3%)	484 (13.0%)	0.934
	Paralogues	340	307	265	645	329	424	393	297	468	274	3,742	
Arachis monticola A	All converted	4 (21.1%)	2 (5.9%)	4 (16.7%)	1 (4.3%)	4 (57.1%)	2 (5.7%)	2 (25.0%)	4 (19.0%)	1 (9.1%)	2 (12.5%)	26 (13.1%)	2.20×10^{-16}
	Paralogues	19	34	24	23	7	35	8	21	11	16	198	
Arachis monticola B	All converted	3 (17.6%)	5 (15.2%)	4 (16.0%)	4 (17.4%)	0 (0.0%)	4 (11.1%)	0 (0.0%)	3 (12.5%)	2 (18.2%)	3 (18.8%)	28 (14.1%)	4.04×10^{-5}
	Paralogues	17	33	25	23	6	36	7	24	11	16	198	
Arachis hypogaea A (Shitouqi)	All converted	31 (11.2%)	22 (12.4%)	49 (8.8%)	28 (14.4%)	27 (8.4%)	24 (9.1%)	13 (10.2%)	38 (12.8%)	9 (4.6%)	11 (5.0%)	252 (9.6%)	0.389
	Paralogues	278	177	556	194	321	265	127	296	195	219	2,628	
Arachis hypogaea B (Shitouqi)	All converted	36 (13.6%)	19 (9.9%)	46 (7.7%)	21 (10.3%)	23 (7.2%)	18 (6.8%)	28 (14.9%)	17 (7.8%)	6 (3.1%)	16 (8.9%)	230 (8.8%)	0.238
	Paralogues	265	192	599	204	321	265	188	219	195	180	2,628	
Arachis hypogaea A (Tifrunner)	All converted	36 (8.4%)	25 (10.0%)	31 (4.2%)	22 (8.0%)	38 (7.6%)	40 (9.3%)	11 (4.4%)	21 (4.5%)	23 (8.4%)	17 (5.6%)	264 (6.8%)	0.738
	Paralogues	431	251	733	274	499	430	248	466	273	301	3,906	
Arachis hypogaea B (Tifrunner)	All converted	30 (7.9%)	20 (8.0%)	46 (6.3%)	21 (7.4%)	44 (8.8%)	31 (7.2%)	22 (7.0%)	24 (6.1%)	25 (9.0%)	15 (4.4%)	278 (7.1%)	0.985
	Paralogues	381	250	735	282	499	430	313	396	277	343	3,906	

Supplemental Table S7. The conversion rate of PRT-related duplicated genes and physical location of genes on chromosomes.

Species		Chr1	Chr2	Chr3	Chr4	Chr5	Chr6	Chr7	Chr8	Chr9	Ch10	All	P-value
Arachis monticola A	All converted	56 (14.9%)	30 (14.9%)	45 (16.2%)	52 (21.1%)	24 (18.5%)	47 (16.5%)	37 (19.0%)	46 (13.6%)	51 (19.7%)	45 (15.0%)	433 (16.5%)	0.952
	Homologous gene	377	201	277	246	130	285	195	337	259	300	2,625	
Arachis monticola B	All converted	52 (16.6%)	31 (16.1%)	44 (14.2%)	52 (17.1%)	24 (22.9%)	47 (16.3%)	51 (20.6%)	32 (11.6%)	51 (18.4%)	48 (15.4%)	433 (16.5%)	0.802
	Homologous gene	313	192	309	304	105	289	248	275	277	312	2,625	
Arachis hypogaea A (Shitouqi)	All converted	98 (8.4%)	71 (10.7%)	258 (16.0%)	72 (9.5%)	111 (10.3%)	107 (11.0%)	71 (10.8%)	123 (12.9%)	128 (11.7%)	83 (8.2%)	1,122 (11.3%)	0.894
	Homologous gene	1,172	665	1,613	756	1,073	972	656	952	1,095	1,018	9,972	
Arachis hypogaea B (Shitouqi)	All converted	90 (8.5%)	77 (11.5%)	271 (15.0%)	74 (9.5%)	108 (10.4%)	107 (11.0%)	101 (11.6%)	85 (11.8%)	133 (11.6%)	76 (8.3%)	1,122 (11.3%)	0.961
	Homologous gene	1,059	671	1,804	775	1,040	972	868	722	1,149	912	9,972	
Arachis hypogaea A (Tifrunner)	All converted	97 (7.3%)	199 (22.8%)	175 (8.8%)	206 (8.4%)	383 (17.1%)	201 (15.0%)	144 (16.1%)	106 (8.4%)	119 (9.3%)	76 (6.9%)	1,706 (13.5%)	1.01×10^{-2}
	Homologous gene	1,330	873	1,991	1,203	1,463	1,343	892	1,262	1,275	1,104	12,656	
Arachis hypogaea B (Tifrunner)	All converted	88 (7.3%)	199 (23.3%)	175 (9.1%)	210 (17.1%)	383 (26.2%)	201 (15.0%)	94 (8.2%)	154 (15.7%)	121 (9.3%)	81 (6.7%)	1,706 (13.5%)	3.06×10^{-4}
	Homologous gene	1,200	854	1,926	1,225	1,463	1,343	1,147	978	1,303	1,217	12,656	

Supplemental Table S8. The converted duplicates of LCT-related in each interval from the terminal.

The rate to the end of the chromosome	0-0.05[a]	0.05-0.1	0.1-0.15	0.15-0.2	0.2-0.25	0.25-0.3	>0.3	P-value	All
Arachis duranensis	146 (33.2%)	90 (20.4%)	56 (12.7%)	43 (9.8%)	28 (6.4%)	17 (3.9%)	60 (13.6%)	2.20×10^{-16}	440
Arachis ipaensi	183 (37.8%)	105 (21.7%)	72 (14.9%)	51 (10.5%)	29 (6.0%)	17 (3.5%)	27 (5.6%)	2.20×10^{-16}	484
Arachis monticola A	9 (34.6%)	1 (3.8%)	3 (11.5%)	1 (3.8%)	2 (7.7%)	3 (11.5%)	7 (26.9%)	1.70×10^{-2}	26
Arachis monticola B	7 (25.0%)	7 (25.0%)	1 (3.6%)	6 (21.4%)	5 (17.9%)	2 (7.1%)	0 (0.0%)	4.30×10^{-2}	28
Arachis hypogaea A (Shitouqi)	46 (18.3%)	52 (20.1%)	43 (17.1%)	42 (16.7%)	20 (7.9%)	16 (6.3%)	33 (13.1%)	2.86×10^{-5}	252
Arachis hypogaea B (Shitouqi)	66 (28.7%)	59 (25.7%)	35 (15.2%)	23 (10.0%)	19 (8.3%)	13 (5.7%)	15 (6.5%)	1.42×10^{-15}	230
Arachis hypogaea A (Tifrunner)	72 (27.3%)	56 (21.2%)	46 (17.4%)	28 (10.6%)	16 (6.1%)	11 (4.2%)	35 (13.3%)	2.41×10^{-14}	264
Arachis hypogaea B (Tifrunner)	116 (41.7%)	47 (16.9%)	29 (10.4%)	29 (10.4%)	21 (7.6%)	17 (6.1%)	19 (6.8%)	2.20×10^{-16}	278

Note: [a]Within 5% of the end of the chromosome.

Supplemental Table S9. The converted duplicates of PRT-related in each interval from the terminal.

The rate to the end of the chromosome	0-0.05	0.05-0.1	0.1-0.15	0.15-0.2	0.2-0.25	0.25-0.3	>0.3	P-value	All
Arachis monticola A	114 (26.3%)	117 (27.0%)	48 (11.1%)	32 (7.4%)	31 (7.2%)	26 (6.0%)	65 (15.0%)	2.20×10^{-16}	433
Arachis monticola B	85 (19.6%)	59 (13.6%)	61 (14.1%)	85 (19.6%)	64 (14.5%)	25 (5.8%)	55 (12.7%)	4.23×10^{-7}	433
Arachis hypogaea A (Shitouqi)	365 (32.5%)	271 (24.1%)	142 (12.7%)	102 (9.1%)	77 (6.9%)	46 (4.1%)	119 (10.6%)	2.20×10^{-16}	1,122
Arachis hypogaea B (Shitouqi)	327 (29.1%)	330 (29.4%)	168 (15.0%)	110 (9.8%)	80 (7.1%)	45 (4.0%)	62 (5.5%)	2.20×10^{-16}	1,122
Arachis hypogaea A (Tifrunner)	975 (57.2%)	272 (15.9%)	132 (7.7%)	93 (5.5%)	74 (4.3%)	48 (2.8%)	112 (6.6%)	2.20×10^{-16}	1,706
Arachis hypogaea B (Tifrunner)	1,064 (62.4%)	246 (14.4%)	139 (8.1%)	93 (5.5%)	71 (4.2%)	47 (2.8%)	46 (2.7%)	2.20×10^{-16}	1,706

Supplemental Table S10. Relationship between the gene physical location and gene conversion of LCT-related.

Species	Distance to telomere	<5 Mb	5-10Mb	10-15Mb	15-20Mb	>20Mb	All
	All converted	135	80	66	58	91	440
Arachis duranensis	Paralogues	1,052	759	594	506	831	3,742
	Mean converted rate	13.10%	10.47%	11.04%	13.43%	8.07%	11.80%
	All converted	145	102	67	52	118	484
Arachis ipaensi	Paralogues	1,211	712	485	388	946	3,742
	Mean converted rate	11.95%	14.32%	13.68%	14.10%	10.38%	13.00%
	All converted	2	4	1	3	16	26
Arachis monticola A	Paralogues	21	16	31	14	106	198
	Mean converted rate	14.48%	8.57%	13.43%	10.83%	8.05%	13.10%
	All converted	0	3	4	1	19	28
Arachis monticola B	Paralogues	10	6	16	10	151	198
	Mean converted rate	31.67%	27.33%	18.57%	4.00%	6.49%	14.10%
	All converted	42	50	43	45	72	252
Arachis hypogaea A (Shitouqi)	Paralogues	546	588	449	348	697	2,628
	Mean converted rate	7.76%	8.54%	9.47%	12.12%	8.94%	9.60%
	All converted	47	49	31	25	78	230
Arachis hypogaea B (Shitouqi)	Paralogues	607	441	367	360	853	2,628
	Mean converted rate	7.69%	10.56%	8.35%	7.72%	7.75%	8.80%
	All converted	59	51	52	35	67	264
Arachis hypogaea A (Tifrunner)	Paralogues	1,161	761	595	458	931	3,906
	Mean converted rate	5.18%	6.69%	8.52%	7.71%	9.17%	6.80%
	All converted	87	43	33	21	94	278
Arachis hypogaea B (Tifrunner)	Paralogues	1,235	702	514	415	1,040	3,906
	Mean converted rate	7.12%	6.00%	6.39%	4.85%	7.69%	7.10%

Supplemental Table S11. Relationship between the gene physical location and gene conversion of PRT-related.

Species	Distance to telomere	<5 Mbp	5-10Mbp	10-15Mbp	15-20Mbp	>20Mbp	All
	All converted	122	113	62	35	101	433
Arachis monticola A	Homologous gene	682	685	350	291	617	2,625
	Mean converted rate	18.04%	16.25%	16.85%	12.57%	12.21%	16.50%
	All converted	51	75	25	39	243	433
Arachis monticola B	Homologous gene	269	381	254	285	1,436	2,625
	Mean converted rate	18.43%	19.78%	9.30%	14.25%	15.75%	16.50%
	All converted	316	294	168	106	238	1122
Arachis hypogaea A (Shitouqi)	Homologous gene	2,390	2,372	1,699	1,101	2,410	9,972
	Mean converted rate	13.26%	12.30%	9.74%	9.75%	8.53%	11.30%
	All converted	225	239	216	106	336	1,122
Arachis hypogaea B (Shitouqi)	Homologous gene	2,185	1,748	1,550	1,097	3,392	9,972
	Mean converted rate	10.32%	13.64%	14.05%	9.66%	8.50%	11.30%
	All converted	883	315	170	107	231	1,706
Arachis hypogaea A (Tifrunner)	Homologous gene	3,794	2,807	2,031	1,234	2,790	12,656
	Mean converted rate	22.48%	10.87%	8.61%	8.79%	6.17%	13.50%
	All converted	892	280	144	103	287	1,706
Arachis hypogaea B (Tifrunner)	Homologous gene	3,741	2,491	1,673	1,236	3,515	12,656
	Mean converted rate	22.96%	11.18%	8.56%	8.33%	6.73%	13.50%

Supplemental Table S12. Relationship between the block number and gene conversion of LCT-related.

Species		Chr1	Chr2	Chr3	Chr4	Chr5	Chr6	Chr7	Chr8	Chr9	Ch10
Arachis duranensis	CV rate	0.14	0.10	0.10	0.13	0.10	0.12	0.14	0.12	0.13	0.12
	Block num	94	41	125	51	66	56	42	82	41	66
Arachis ipaensi	CV rate	0.17	0.12	0.11	0.11	0.14	0.13	0.15	0.14	0.10	0.15
	Block num	127	59	63	146	57	61	66	83	57	34
Arachis monticola A	CV rate	0.21	0.06	0.17	0.04	0.57	0.06	0.25	0.19	0.09	0.13
	Block num	80	23	40	57	50	32	32	28	23	46
Arachis monticola B	CV rate	0.18	0.15	0.16	0.17	0.00	0.11	0.00	0.13	0.18	0.19
	Block num	39	29	85	28	18	50	75	58	23	58
Arachis hypogaea A (Shitouqi)	CV rate	0.11	0.12	0.09	0.14	0.08	0.09	0.10	0.13	0.05	0.05
	Block num	87	40	107	35	62	60	47	82	47	63
Arachis hypogaea B (Shitouqi)	CV rate	0.14	0.10	0.08	0.10	0.07	0.07	0.15	0.08	0.03	0.09
	Block num	42	41	109	83	51	104	84	26	50	39
Arachis hypogaea A (Tifrunner)	CV rate	0.08	0.10	0.04	0.08	0.08	0.09	0.04	0.05	0.08	0.06
	Block num	78	38	127	47	62	53	46	75	55	51
Arachis hypogaea B (Tifrunner)	CV rate	0.08	0.08	0.06	0.07	0.09	0.07	0.07	0.06	0.09	0.04
	Block num	70	37	113	52	72	83	106	53	42	89

Supplemental Table S13. Relationship between the block number and gene conversion of PRT-related.

Species		Chr1	Chr2	Chr3	Chr4	Chr5	Chr6	Chr7	Chr8	Chr9	Ch10
Arachis monticola A	CV rate	0.15	0.15	0.16	0.21	0.19	0.17	0.19	0.14	0.20	0.15
	Block num	80	23	40	57	50	32	32	28	23	46
Arachis monticola B	CV rate	0.17	0.16	0.14	0.17	0.23	0.16	0.21	0.12	0.18	0.15
	Block num	39	29	85	28	18	50	75	58	23	58
Arachis hypogaea A (Shitouqi)	CV rate	0.08	0.11	0.16	0.10	0.10	0.11	0.11	0.13	0.12	0.08
	Block num	87	40	107	35	62	60	47	82	47	63
Arachis hypogaea B (Shitouqi)	CV rate	0.09	0.12	0.15	0.10	0.10	0.11	0.12	0.12	0.12	0.08
	Block num	42	41	109	83	51	104	84	26	50	39
Arachis hypogaea A (Tifrunner)	CV rate	0.07	0.23	0.09	0.08	0.17	0.15	0.16	0.08	0.09	0.07
	Block num	78	38	127	47	62	53	46	75	55	51
Arachis hypogaea B (Tifrunner)	CV rate	0.07	0.23	0.09	0.17	0.26	0.15	0.08	0.16	0.09	0.07
	Block num	70	37	113	52	72	83	106	53	42	89

Supplemental Table S14. Relationship between the block length (colinear gene pairs) and gene conversion of LCT-related.

Block length		0-10	10-20	20-30	30-40	40-50	>50
Arachis duranensis	CV rate	0.04	0.06	0.098	0.098	0.097	0.135
Arachis ipaensi	CV rate	0.038	0.071	0.059	0.081	0.108	0.104
Arachis monticola A	CV rate	0.006	0.014	0	-	0	-
Arachis monticola B	CV rate	0.005	0.008	0	-	0	-
Arachis hypogaea A (Shitouqi)	CV rate	0.033	0.035	0.093	0.098	0.102	0.099
Arachis hypogaea B (Shitouqi)	CV rate	0.026	0.046	0.034	0.093	0	0.057
Arachis hypogaea A (Tifrunner)	CV rate	0.033	0.037	0.045	0.084	0.056	0.053
Arachis hypogaea B (Tifrunner)	CV rate	0.025	0.050	0.057	0.060	0.093	0.067

Supplemental Table S15. Relationship between the block length (colinear gene pairs) and gene conversion of PRT-related.

Block length		0-50	50-100	100-150	150-200	>200
Arachis monticola	CV rate	0.053	0.097	0.108	-	0.123
Arachis hypogaea (Shitouqi)	CV rate	0.036	0.070	0.088	0.070	0.081
Arachis hypogaea (Tifrunner)	CV rate	0.044	0.075	0.230	0.270	0.092

Supplemental Table S16. Statistics of the converted and duplicated genes from top four functions from the Shitouqi and Tifrunner genomes.

**GO level2**	*A. hypogaea* A (Shitouqi)		*A. hypogaea* B (Shitouqi)		*A. hypogaea*A (Tifrunner)		*A. Hypogaea* B (Tifrunner)	
	**cv *vs.* all**	**P-value**	**cv *vs.* all**	**P-value**	**cv *vs.* all**	**P-value**	**cv *vs.* all**	**P-value**
Catalytic activity	31.4 *vs.* 25.5	0.802	20.4 vs. 15.1	0.029	23.3 vs. 15.1	<0.001	19.4 vs. 15.4	0.088
Binding	31.7 *vs.* 28.2	0.323	17.4 vs. 18.7	0.609	21.0 vs. 19.5	0.543	19.6 vs. 19.1	0.820
Metabolic process	25.7 *vs.* 23.7	0.482	18.3 vs. 15.6	0.281	24.8 vs. 15.2	<0.001	18.2 vs. 15.1	0.174
Cellular process	17.5 *vs.* 19.6	0.550	15.7 vs. 12.4	0.151	14.5 *vs.* 12.3	0.294	10.5 *vs.* 12.1	0.454

Note: Percentage of converted genes (cv), percentage of duplicated genes (all).
